# The impact of parental touch and companionship on the emergence period of paediatric snoring surgery under general anaesthesia

**DOI:** 10.1017/S0022215126104289

**Published:** 2026-05

**Authors:** Yi fang Wang, Chengyan Zhong, Yue Liu

**Affiliations:** 1 Department of Anesthesiology II, The First People’s Hospital of Chuzhou (The Affiliated Chuzhou Hospital of Anhui Medical University), Anhui Province, China; 2 Department of Anesthesiology and Surgery, Children’s Hospital, the First People’s Hospital of Chuzhou City, Chuzhou, China

**Keywords:** Parents, Peri-operative Period, Anaesthesia, General, Snoring, Surgical Procedures, Operative, Child

## Abstract

**Objectives:**

To explore the effect of parental presence on the recovery period of paediatric patients undergoing general anaesthesia for snoring surgery.

**Methods:**

Forty-two paediatric patients who underwent snoring surgery were randomly divided into control and observation groups. The control group received routine nursing intervention, while the observation group allowed either the father or mother to enter the recovery room immediately after the child was awake and extubated, in addition to the routine nursing intervention.

**Results:**

Paediatric anaesthesia emergence delirium score during the recovery period was significantly lower in the observation group than the control group. The Face, Legs, Activity, Cry, Consolability score during the recovery period was also significantly lower in the observation group. Parental satisfaction with nursing was significantly higher in the observation group.

**Conclusion:**

Immediate parental presence can effectively alleviate insecurity, reduce pain, decrease crying and restlessness of paediatric patients, and improve parental satisfaction with nursing.

## Introduction

Adenotonsillectomy is a common surgical procedure for the treatment of paediatric obstructive sleep-disordered breathing. However, emergence agitation (EA) is a frequent complication during recovery from general anaesthesia, characterised by crying, thrashing, disorientation and other agitated behaviours. Emergence agitation not only complicates post-operative care but may also lead to adverse events such as airway obstruction and surgical wound bleeding.^1^^,^^2^ Studies indicate that the incidence of EA in children can reach 48 per cent.^2^ The underlying mechanisms may involve residual effects of anaesthetic agents (e.g., sevoflurane), post-operative pain, unfamiliar environments and anxiety due to separation from parents.^1^^,^^3^ Currently, pharmacological interventions, such as dexmedetomidine or ketamine-based combinations, are commonly used to mitigate EA.^3^^–^^5^ However, these agents may cause side effects, including respiratory depression and delayed emergence.^4^^,^^6^ Therefore, exploring non-pharmacological intervention strategies is of great clinical significance.

Parental presence has gained increasing attention as an effective measure to reduce peri-operative anxiety in children. Multiple studies have demonstrated that parental presence during induction of anaesthesia (PPIA) can reduce pre-operative anxiety levels.^7^^,^^8^ Nevertheless, its impact on the emergence phase remains controversial. Some researchers suggest that parental presence may reduce the incidence of EA by alleviating separation anxiety and enhancing the child’s sense of security.^9^ In contrast, other studies indicate that highly anxious parents may heighten their child’s perception of pain and increase the risk of post-operative complications.^10^ These conflicting results suggest that the timing, manner and psychological state of parents may critically influence the intervention outcome. One randomised controlled trial found that repetitive maternal voice guidance significantly reduced the incidence of EA,^9^ while another study emphasised that allowing the child to choose which parent accompanies them may be more effective in reducing post-operative delirium than having the parent unilaterally decide.^8^

Furthermore, post-operative pain is a significant trigger for EA.^11^ Pain assessment tools such as the Face, Legs, Activity, Cry, Consolability (FLACC) scale have shown that children often experience moderate to severe pain after adenotonsillectomy, which is significantly correlated with internalising problems in parents (e.g., anxiety and need for information).^11^ High parental anxiety may exacerbate the child’s pain experience through emotional contagion, leading to higher complication rates (e.g., 31.4 per cent–57.1 per cent).^10^ Therefore, while implementing parental presence, it is essential to assess and support the psychological state of parents. Previous research has primarily focused on PPIA or used audiovisual interventions (e.g., video demonstrations or voice guidance) to indirectly simulate parental presence.^9^^,^^12^ Few studies have investigated the immediate effects of direct parental touch and companionship during the emergence phase. Against this background, this study focuses on parental presence immediately after extubation during emergence and examines its effects on EA, pain levels and family satisfaction.

## Materials and methods

### Subjects

This study was approved by the Ethics Committee of the Chuzhou First People’s Hospital. All guardians of the paediatric patients signed informed consent forms. A total of 42 paediatric patients who underwent snoring surgery at Chuzhou First People’s Hospital from January 2024 to March 2025 were included. They underwent tonsil and/or adenoid removal under general anaesthesia with endotracheal intubation. They were randomly divided into a control group and an observation group based on the intervention method during the anaesthesia recovery period (*n* = 21). Inclusion criteria: (1) elective tonsillectomy and adenoidectomy under general anaesthesia; (2) age between 3 and 12 years old; (3) pre-anaesthesia condition and physical status meeting the criteria of American Society of Anesthesiologists (ASA) classification I–II^4^; (4) assessment of the child’s condition 1 day before surgery, with clear consciousness and normal hearing and language expression ability. Exclusion criteria: (1) combination with severe systemic diseases and developmental abnormalities, unable to co-operate with the study; (2) presence of mental illness and severe cognitive impairment in the child and their parents, unable to communicate effectively; (3) refusal of participation by family members or absence of a suitable companion.

### General anaesthesia protocol

A standardised general anaesthesia protocol (maintained with propofol-remifentanil) was employed during surgery, along with fixed-dose fentanyl administration according to institutional guidelines. Post-operative analgesia was provided using a weight-based standardised rescue regimen (intravenous morphine patient-controlled analgesia (PCA)). Both groups received consistent protocols to control for confounding variables. Anesthesiologists were not blinded due to the need to implement different anaesthesia protocols. The standardised anaesthesia protocol specifically pertains to the uniformity of intra-operative anaesthesia management (selection and dosage of anaesthetic drugs), whereas the post-anaesthesia care unit (PACU) management protocols differed between the groups. The non-blinded design for the anaesthesia team was based on the following considerations: (1) the need for safety monitoring (parental presence may influence the child’s physiological status); (2) the requirement for precise execution of procedures (accurate co-ordination of extubation timing and parental entry); and (3) compliance with the ASA guidelines that prioritise patient safety.

### Post-operative care for the control group

The paediatric patients in the control group underwent routine nursing interventions, which are primarily described as follows. Firstly, rigorous handover procedures were conducted with circulating nurses and anaesthesiologists, specifically including the exchange of information regarding intra-operative changes in vital signs, anaesthetic medications and anaesthesia methods. Secondly, careful monitoring of the patient’s vital signs was carried out. Thirdly, emphasis was placed on positional nursing interventions to ensure unobstructed airways and prevent complications such as vomiting, aspiration, laryngeal oedema, laryngeal spasms and tongue retroversion. Furthermore, psychological nursing interventions for the patients were strengthened. Depending on the age of the patients, verbal or non-verbal methods were utilised to provide comfort, enhance their co-operation and compliance, and reduce the incidence of restlessness.

### Post-operative care for the observation group

In the observation group, based on the control group, the parent presence recovery programme (PPRP) service was added, with the following main practices. Firstly, recovery room nurses provided educational guidance to the children and their accompanying parents before surgery. Simultaneously, the children were allowed to choose one of their parents to accompany them to the recovery room after surgery. Secondly, targeted training was provided to the accompanying parents, instructing them on how to control their own negative emotions when seeing the child after surgery. Additionally, standardised comfort training was offered, including the use of specific comforting phrases, touching the child’s forehead and holding one of the child’s hands, while refraining from using frightening language. Thirdly, the timing of the child’s family member accompaniment was established. Once the paediatric patient had been extubated, regained spontaneous respiration and demonstrated responsiveness to verbal stimuli, the pre-selected family members were immediately notified to enter the resuscitation room to accompany the child. The attire of the child’s family member complied with the operating room management regulations.

### Paediatric anaesthesia emergence delirium scoring

The paediatric patients’ restlessness during anaesthesia recovery was evaluated using the paediatric anaesthesia emergence delirium (PAED) score.^13^ The PAED scoring criteria are shown in Table 1. The sum of the five scores constitutes the recovery period restlessness score. A higher score indicates a greater likelihood and severity of restlessness. A score greater than 16 is diagnostic of restlessness during the recovery period.

**Table 1.** The PAED scoring criteria Table 1 long description.

Items for child patient	4 points	3 points	2 points	1 point	0 point
Following instructions and communicating	Not at all	Only a little	Some	Many	Extremely many
Purposeful behaviour	Not at all	Only a little	Some	Many	Extremely many
Attentive to the surrounding environment	Not at all	Only a little	Some	Many	Extremely many
Restless	Extremely many	Many	Some	Only a little	
Crying inconsolably	Extremely many	Many	Some	Only a little	

### Face, legs, activity, cry, consolability scoring

For paediatric pain assessment, the FLACC score was chosen (Table 2).^14^ This scoring system is the preferred method for evaluating post-operative pain in children and is commonly used for this purpose. The score ranges from 0 to 10, with higher values indicating greater pain severity.

**Table 2.** The FLACC scoring criteria Table 2 long description.

Items	0	1	2
Face	Neutral or smiling expression	Occasionally displaying a painful expression, frowning, and unwilling to communicate	Frequent chin trembling or tight lower lip biting
Leg	Relaxed or adopting a usual posture	Appearing restless, tense, and maintaining an uncomfortable posture	Kicking or dragging of legs
Activity	Lying quietly, in a normal position, or engaging in gentle activity	Twisting, tossing, and turning, exhibiting tension	Bodily convulsions, arching, and stiffness
Cry	Not crying (whether awake or asleep)	Groaning, occasionally complaining	Constant crying, screaming, and frequent complaints of pain
Consolability	Content and relaxed	Can be comforted by occasional touching, hugging, or words	Difficult to comfort

### Survey on nursing satisfaction among family members of paediatric patients

Using our hospital’s self-developed nursing satisfaction survey, we surveyed the nursing satisfaction of the family members of the two groups of paediatric patients. The survey included aspects such as the service attitude towards the patients, the concept of caring for the injured, the guidance skills for the patient’s discomfort and the anaesthesia nurses’ mastery of disease knowledge. The satisfaction levels were rated as follows: ‘Satisfied’ corresponding to a score of 90–100; ‘Basically Satisfied’ with a score of 70–89; and ‘Dissatisfied’ with a score of less than or equal to 69.

### Statistical analysis

Statistical analysis was performed using SPSS (version 25.0; IBM, Armonk, New York, USA). Measurement data were expressed as mean ± standard deviation (SD). Independent sample *t*-tests were used for comparisons between groups, while paired sample *t*-tests were employed for within-group comparisons. Repeated measures ANOVA and multiple comparisons were applied to analyse the results of multiple repeated measurements. All statistical data were considered statistically significant at *p* < 0.05.

## Results

### The general clinical information in the two groups

In the control group, there were 9 females and 12 males. Their average age was 9.19 ± 2.18 years old, ranging from 5 to 12 years old. In the observation group, there were 10 females and 11 males. Their average age was 7.38 ± 2.42 years old, ranging from 3 to 12 years old. There was no significant difference in gender and age between the observation group and the control group (*p* > 0.05). Moreover, there was no significant difference in body weight between the control and observation groups (46.95 ± 17.11 vs. 32.73 ± 11.87; *p* > 0.05). The control group consisted of 15 children with tonsillar hypertrophy accompanied by adenoid hypertrophy and 6 children with adenoid hypertrophy. The observation group included 17 children with tonsillar hypertrophy accompanied by adenoid hypertrophy and 4 children with adenoid hypertrophy.

### Comparison of paediatric anaesthesia emergence delirium scores between the two groups

**Figure 1.**
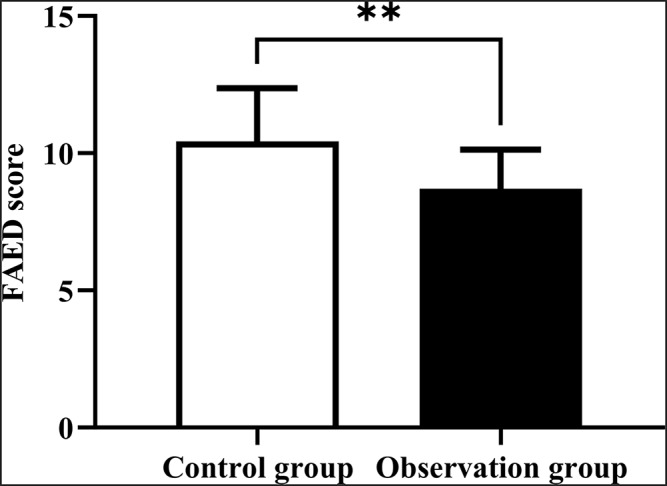
Comparison of PAED scores between the two groups. Figure 1 long description.

The PAED score during the recovery period was 8.706 ± 1.425 points in the observation group (Figure 1). The PAED score during the recovery period was 10.429 ± 1.936 points in the control group (Figure 1). The PAED score in the observation group was significantly lower compared to the control group (*p* < 0.05).

### Comparison of face, legs, activity, cry, consolability scores between the two groups

**Figure 2.**
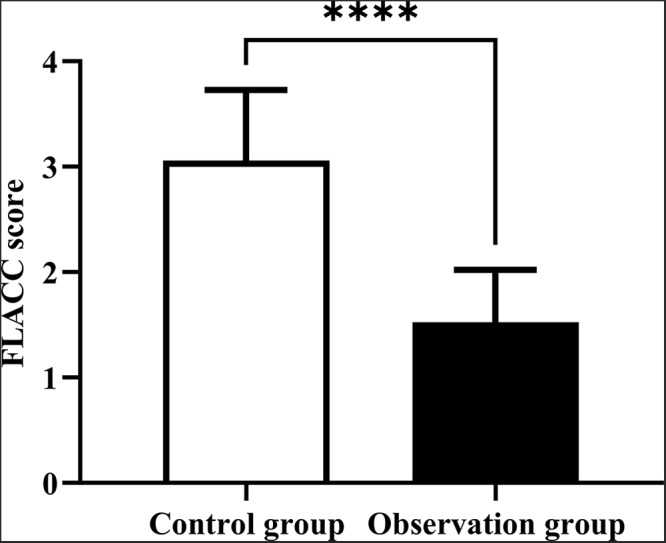
Comparison of FLACC scores between the two groups. Figure 2 long description.

The FLACC score during the recovery period was 1.524 ± 0.496 points in the observation group (Figure 2). The FLACC score during the recovery period was 3.057 ± 0.672 points in the control group (Figure 2). The FLACC score in the observation group was significantly lower compared to the control group (*p* < 0.05).

### Comparison of parental satisfaction with nursing between the two groups

In the control group, there were six dissatisfied cases, whereas in the observation group, there was only one dissatisfied case (Table 3). Parental satisfaction with nursing was significantly higher in the observation group compared to the control group (95.23 per cent vs. 71.42 per cent; *p* < 0.05).

**Table 3.** Comparison of parental satisfaction with nursing between the two groups Table 3 long description.

Groups	Satisfaction	General satisfaction	Non- satisfaction	Satisfaction rate (%)
Control	7	9	7	71.42
Observation	16	6	1	95.23

## Discussion

Paediatric snoring disorder is a common clinical condition often treated with tonsillectomy and/or adenoidectomy to alleviate its symptoms.^15^ However, due to children’s young age, immature psychological development, poor pain tolerance, and the shared location of the surgical site and endotracheal intubation, the combination of surgical trauma and pressure from the endotracheal tube can exacerbate pharyngeal pain after awakening.^16^ This often leads to increased crying and restlessness among paediatric patients. Post-operative emergence agitation (PEA) is a frequent occurrence during the recovery phase of anaesthesia, particularly in children, with an incidence rate ranging from approximately 10 per cent to 67 per cent.^17^

Notably, the incidence of EA after general anaesthesia in otolaryngology surgeries is higher compared to other surgical procedures.^18^ This agitation may manifest as crying, struggling, disorientation or aggressive behaviour. Although typically brief (lasting from 5 to 15 minutes), it can pose various risks, such as the dislodging of monitoring devices (e.g., pulse oximetry probes, electrocardiogram (ECG) leads) during the emergence from anaesthesia, delaying the detection of abnormal vital signs.^19^ Additionally, the dislodging of intravenous lines or drug extravasation can affect post-operative analgesia and fluid replacement therapy.^18^ Post-operative EA can also prolong the recovery time, sometimes requiring the administration of additional sedative drugs (such as benzodiazepines or dexmedetomidine), which may induce complications like respiratory depression and delayed awakening.^20^ Furthermore, it extends the stay in the PACU, increasing the consumption of medical resources.^21^

The results of this study demonstrate that there were no significant differences in baseline data, such as gender and age, between the observation group and the control group, indicating that the two groups were comparable in demographic characteristics, effectively reducing the interference of confounding factors on the results. It is worth noting that despite the consistent age distribution between the two groups, the proportion of cases with adenoid hypertrophy combined with tonsillar hypertrophy in the observation group (17 cases) was slightly higher than that in the control group (15 cases), while the proportion of cases with isolated adenoid hypertrophy was slightly lower (4 cases vs. 6 cases). Although not statistically significant, such subtle differences may reflect heterogeneity in clinical phenotypes.

In this study, the implementation of parental presence during PPRP resulted in significantly lower PAED and FLACC scores in the observation group compared to the control group. The presence of a familiar caregiver allowed the children to see their loved ones immediately upon emerging from anaesthesia, experience their affectionate touch and hear familiar voices, thereby alleviating their fear of the unfamiliar environment.^22^ This companionship served to distract the children, providing emotional support that mitigated their pain. It has been reported that physical touch can stimulate the brain to secrete encephalin, which has analgesic and sedative effects.^23^ Additionally, it can excite cutaneous tactile receptors, promoting the secretion of acetylcholine, which in turn lowers heart rate and blood pressure, and reduces sweat secretion.^24^ Furthermore, early parental presence reduced the children’s exposure to stressful stimuli in an unfamiliar environment.^25^ The children’s needs and concerns could be communicated to their caregivers, who then provided timely feedback to the anaesthesia nursing team for appropriate responses based on specific situations.^26^

This study also indicates a significant increase in the satisfaction of the relatives of the children in the observation group towards nursing care, with a satisfaction rate of 95.83 per cent. This improvement may be attributed to the fact that children were able to see their loved ones immediately after waking up from anaesthesia and extubation, which provided comfort to both the children and their families.^27^ Additionally, the active participation of parents in the nursing process and the fulfilment of their right to know may have also contributed to the high satisfaction rate.^28^ The involvement and companionship of the families can alleviate the workload of nurses, ensuring the effective completion of nursing tasks.^29^

This study has several limitations. Firstly, all data were derived from a single medical institution, which may be subject to regional diagnostic and treatment norms or patient characteristics, thereby affecting the extrapolation of results. Secondly, the study only focused on short-term effects during the recovery period and lacked long-term follow-up data (such as post-operative complications or long-term quality of life), making it impossible to evaluate the sustained effects of the intervention. Future research should expand the sample size, adopt a multicentre design and incorporate long-term follow-up data to enhance the reliability of the findings.

## Conclusion

In conclusion, the presence of family members during the extubation and recovery period of paediatric tonsillectomy and/or adenoidectomy under general anaesthesia has a positive effect. Immediately after extubation, during the recovery period of paediatric tonsillectomy and/or adenoidectomy under general anaesthesia, the intervention of family members’ presence can effectively alleviate the patients’ sense of insecurity, reduce pain, decrease crying and restlessness, and improve parents’ satisfaction with nursing care.

## Data Availability

The data that support the findings of this study are available from the corresponding author upon reasonable request.

## Table 1 Long description

The table evaluates child patients using the PAED scoring criteria across five behavioral items: following instructions, purposeful behavior, attentiveness, restlessness, and crying. Each item is scored from 0 to 4 points, with higher scores indicating less favorable behavior. For following instructions, purposeful behavior, and attentiveness, 'Not at all' scores 4 points, while 'Extremely many' scores 0 points. Conversely, for restlessness and crying, 'Extremely many' scores 4 points, indicating a negative behavior, while 'Only a little' scores 1 point. This scoring system helps in assessing the severity of post-anesthesia emergence delirium in children, with higher scores suggesting more severe symptoms.

Navigate back to Table 1.

## Table 2 Long description

The FLACC scoring criteria table evaluates pain in children by observing five categories: face, legs, activity, cry, and consolability. Each category is scored from 0 to 2, with 0 indicating no pain and 2 indicating severe pain. For example, a neutral face scores 0, while frequent chin trembling scores 2. Similarly, relaxed legs score 0, while kicking scores 2. Activity ranges from lying quietly (0) to bodily convulsions (2). Crying is scored from not crying (0) to constant crying (2). Consolability ranges from content (0) to difficult to comfort (2). This scoring system helps caregivers assess and manage pain effectively in non-verbal children.

Navigate back to Table 2.

## Figure 1 Long description

A bar graph comparing PAED scores between two groups. The y-axis is labeled 'PAED score' with a range from 0 to 15. The x-axis shows 'Control group' and 'Observation group'. The control group bar reaches slightly above 10, while the observation group bar is slightly below 10. An asterisk above the bars indicates a significant difference between the groups.

Navigate back to Figure 1.

## Figure 2 Long description

A bar graph comparing FLACC scores between two groups. The x-axis is labeled 'Control group' and 'Observation group'. The y-axis is labeled 'FLACC score' ranging from 0 to 4. The control group bar reaches approximately 3, while the observation group bar is lower, around 1.5. Error bars are present on both bars and asterisks indicate a significant difference between the groups.

Navigate back to Figure 2.

## Table 3 Long description

The table compares parental satisfaction with nursing between control and observation groups. The observation group shows a significantly higher satisfaction rate of 95.23%, with 16 parents satisfied, 6 generally satisfied, and only 1 non-satisfied. In contrast, the control group has a satisfaction rate of 71.42%, with 7 parents satisfied, 9 generally satisfied, and 7 non-satisfied. This suggests that the observation group had a more favorable experience, with a notable difference in satisfaction levels between the two groups.

Navigate back to Table 3.
